# Co-Design and Pilot Testing of a Nurse-Led GP-Supported Self-Management Intervention for Breast Cancer Survivors with Cardiovascular Diseases: A Study Protocol

**DOI:** 10.3390/nursrep16070253

**Published:** 2026-07-20

**Authors:** Anu Correya, Jing-Yu (Benjamin) Tan, Xian-Liang Liu, Daniel Bressington, Leah East, Tao Wang

**Affiliations:** 1 School of Nursing and Midwifery, University of Southern Queensland, Toowoomba, QLD 4350, Australia; benjamin.tan@nd.edu.au (J.-Y.T.); leah.east@unisq.edu.au (L.E.); alison.wang@ecu.edu.au (T.W.); 2 Institute of Health and Well-Being, Federation University Australia, Berwick, VIC 3808, Australia; 3 Intensive Care Unit, Monash Health, Clayton, VIC 3168, Australia; 4 School of Nursing and Midwifery, The University of Notre Dame Australia, Fremandle, WA 6160, Australia; 5 School of Nursing and Health Sciences, Hong Kong Metropolitan University, Hong Kong, China; dxliu@hkmu.edu.hk; 6 Faculty of Nursing, Chiang Mai University, Chiang Mai 50200, Thailand; 7 Institute of Health, University of Southern Queensland, Toowoomba, QLD 4350, Australia; 8 School of Health, University of New England, Armidale, NSW 2350, Australia; 9 School of Nursing and Midwifery, Edith Cowan University, Joondalup, WA 6027, Australia

**Keywords:** breast cancer, breast cancer survivors, cardiovascular diseases, self-management, co-design

## Abstract

**Background/Objectives**: Self-management intervention strategies are recommended to address the needs of breast cancer survivors (BCSs) who have cardiovascular diseases (CVDs). Despite the benefits, these strategies are often suboptimally implemented, resulting in inadequate cardiovascular management among BCS with CVDs. The project aims to develop a nurse-led and GP-supported self-management (NGPS) intervention to reduce cardiovascular risks in BCS with CVDs and evaluate its feasibility and potential effects in primary care settings using the double diamond co-design process and the Medical Research Council (MRC) Framework for Developing and Evaluating Complex Interventions. **Methods**: The study will be conducted in two phases. In phase I, an evidence-based and co-designed NGPS protocol with end-users will be preliminarily developed based on the identified research evidence, relevant theories, practice guidelines and the current practice standards in Australia. The co-design workshop(s) will involve healthcare professionals and BCSs with CVDs to further refine and validate the developed preliminary protocol. In phase II, a one-group pre-post pilot study will evaluate the feasibility of the intervention and study procedures, such as recruitment, retention, adherence, acceptability, and safety as primary outcomes. The study will also preliminarily explore the effectiveness of the intervention such as physiological measures as secondary outcomes to inform a future large-scale trial. Phase II also involves follow-up qualitative interviews to explore participants’ experiences of the pre-post pilot study. The participants will be recruited from primary medical centres in Victoria. **Conclusions**: The findings will provide a co-designed evidence-based intervention for primary care nurses and General Practitioners (GPs) to promote long-term health outcomes for patients with both breast cancer (BC) and CVDs. **Clinical registration**: ClinicalTrial.gov (registration No. NCTO7313397).

## 1. Introduction

Breast cancer (BC) is the most commonly diagnosed cancer among women in 157 out of 185 countries in 2022, with a reported 670,000 deaths in 2022 [1]. According to the Australian Institute of Health and Welfare 2023 [2]. BC was the most prevalent type of cancer in females in 2022, representing 28% of all reported female cancers. There is a steady rise in breast cancer survivors (BCSs) because of improvements in screening and treatment advancements [3]. However, BCSs are at high risk of developing cardiovascular diseases (CVDs) due to the cardiotoxic effects of BC treatment as well as the overlapping risk factors between BC and CVDs, such as smoking, alcohol consumption, obesity and hypertension [4,5,6]. Further, BCSs are at up to eight times higher risk of developing CVDs, and up to 25% of BCSs die from CVDs that occur within seven years of the cancer diagnosis, becoming the leading cause of death in this population [7].

Cardio-oncology is a subspecialty with a collaborative team that provides cardiovascular care by identifying, preventing and managing CVDs in cancer patients [8,9]. Cardiac rehabilitation is a non-pharmacological cardio-oncology programme for cancer survivors who are at high risk of CVDs [10]. Numerous studies have reported the use of exercise and diet as part of a cardio-oncology program to prevent cardiotoxicity during BC treatment [11,12]. For example, a randomised controlled trial (RCT) examined the effectiveness of an exercise-based cardio-oncology program in preventing cancer-therapy-related cardiac dysfunction and showed significant reductions in left ventricular ejection fraction and Body Mass Index (BMI) in obese BC patients [12]. Another RCT examined the evidence-based cardioprotective diet in preventing anthracycline-induced cardiomyopathy (AIC) and found that adherence to this diet contributed to a reduced risk of AIC [11]. Although these non-pharmacological cardio-oncology services are available, they are not easily accessible or self-manageable as they are specialist-dependent, hospital-based, and poorly scalable. Poor coordination between cardiology and oncology services and the absence of shared-care models result in fragmented care pathways, delayed diagnosis, and management of cardiovascular complications in cancer patients [13]. Moreover, a recent systematic review identified several challenges in cardio-oncology programmes among BCSs, which included limited availability of programmes, gender-influenced barriers to participation, cost-related limitations, underutilisation of cardiac rehabilitation, delayed initiation of cardiac rehabilitation, physical health limitations, psychosocial difficulties and socioeconomic and racial inequalities [14]. Considering these challenges, self-management interventions could be a great supporting tool to meet the needs of patients with both BC and CVDs [15,16]. Limited studies have been conducted to examine the use of preventive, non-pharmacological, self-manageable cardioprotective strategies to reduce cardiotoxicity in BCSs. This study integrates evidence-based cardiovascular risk reduction strategies with survivorship care recommendations to create a comprehensive approach. The proposed novel intervention will be co-designed with BCSs, healthcare professionals, and researchers, ensuring that the end-users perspectives directly inform its development. To our knowledge, no previous intervention has combined CVD prevention strategies into BC survivorship care while actively involving survivors and healthcare professionals in the development process.

Self-management interventions are multifaceted behavioural interventions to guide patients to implement and reinforce a set of positive health behaviours in day-to-day life [17,18]. Self-management interventions for women with BC include exercise to improve cardiovascular fitness, reduce fatigue and enhance overall Quality of Life (QoL) and adherence to healthy dietary habits to reduce the risk of non-BC death [19,20]. Other research indicated that health behaviour modifications such as a healthy diet and regular physical activity can help reduce the risk of CVDs in BCSs [21]. However, the application of these strategies continues to be limited due to challenges such as restricted access to specialised cardio-oncology services or financial constraints leading to poor management of CVDs in BCSs with CVDs [22,23].

In Australia, primary healthcare professionals are often the patient’s first point of contact within the health system [24]. Nurses and General Practitioners (GPs) play a fundamental role in primary care for the care of BCSs and the effective implementation of the survivorship care plan [25]. Nurses can provide these interventions either face-to-face or by email or by telephone [26]. Previous research pointed out the cost-effectiveness and the cancer patients’ high level of satisfaction after being treated by primary care physicians due to their long-term relationship [27]. However, the existing models of post-treatment cancer care are mainly hospital-based, medical specialists-led, and do not utilise primary care [28]. Evidence from systematic reviews, RCTs, and international guidelines supports the effectiveness of nurse-led interventions in cancer survivorship programmes as they improve QoL, fatigue, and psychosocial outcomes while achieving clinical outcomes comparable to specialist-led care [29,30,31,32]. Nurse-led survivorship care also enhances care coordination, patient satisfaction, and cost-effectiveness, making it a sustainable and patient-centred approach to managing BCSs [29,33]. The role of nurses and GPs in primary care settings and the current gap in the management of chronic diseases highlighted the need for the integration of self-management intervention programmes in primary care settings for BCSs with CVDs.

Our team has previously conducted two systematic reviews to synthesise the existing evidence of self-managed non-pharmacological interventions for BCSs [34] and CVDs [35]. The reviews identified common non-pharmacological interventions for BCSs and CVDs, which could guide the selection and development of an evidence-based non-pharmacological intervention programme for BCSs with CVDs in this research project. Managing two complex health conditions, BC and CVD simultaneously presents distinct challenges such as clinical and system-level challenges that may influence how patients respond to or benefit from self-managed non-pharmacological interventions. Clinical challenges such as shared modifiable and non-modifiable risk factors, cardiotoxic effects of cancer treatments, and poor cancer outcomes due to CVD, and system-level challenges such as poor integration of cardio-oncology services, lack of coordination between specialties, absence of a shared care model and limited awareness among clinicians about cardiotoxicity complicate the long-term surveillance of BCSs [4,28,36,37]. Considering these challenges and the elevated risk for CVDs, this highlights an urgent need for tailored, evidence-based self-management interventions delivered within primary healthcare.

This research project was thus proposed to develop a nurse-led and GP-supported self-management (NGPS) intervention for BCSs with CVDs and evaluate its feasibility and potential effects in primary care settings with the following specific objectives: (1) To develop an evidence based NGPS intervention protocol that is tailored to the needs of BCS with CVDs; (2) To examine the feasibility, acceptability, and safety of the NGPS intervention in BCS with CVDs; (3) To test the preliminary effects of the NGPS intervention on cardiovascular health outcomes, such as physiological outcomes, behavioural outcomes including dietary intake, physical activities and smoking, psychosocial outcomes including self-efficacy, QoL, anxiety and depression, and healthcare usage, in BCS with CVDs; (4) To explore the experiences of patients and healthcare professionals who participate in this pilot study and to identify any barriers and facilitators in the implementation of the NGPS intervention in Australian primary care settings; and (5) To refine the NGPS protocol based on the results of this pilot trial and the qualitative process evaluation.

## 2. Materials and Methods

Overall research design

**Figure 1:**
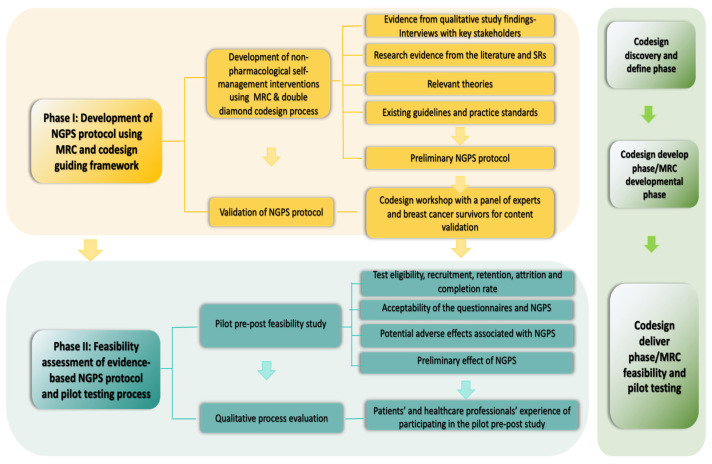
Overall research design.

A multi-method study design will be used. The project will adopt phase I and phase II of the updated Medical Research Council (MRC) framework for developing and evaluating complex interventions as a guide [38]. The first two phases, focused on identifying a new or existing intervention and exploring its feasibility, will guide the development and validation of an evidence-based NGPS intervention protocol and evaluate its feasibility by identifying the research evidence, relevant theories, developing and validating the preliminary NGPS protocol and feasibility testing of the developed protocol [38]. Given the limited context-based instructions on intervention development and delivery methods in the MRC framework [39], this research project will incorporate the double diamond co-design approach [40] to structure the NGPS intervention development. Incorporating co-design processes within the MRC framework enhances intervention design by involving diverse perspectives, increasing applicability and facilitating co-development of knowledge in the development phase of the co-design process and will benefit from managing their medical condition [41,42,43]. The overall research design is presented in Figure 1.

### 2.1. Phase I Development of a Preliminary NGPS Protocol Using the MRC Framework and Double Diamond Co-Design Process

Phase I involves the development of a co-designed NGPS protocol with end-users based on the two qualitative study findings, relevant theories, identified research evidence, practice guidelines and current practice standards in Australia using co-design workshop(s) with healthcare professionals and BCSs followed by a content validation study. The protocol will be developed by synthesising ideas and concepts generated from the two qualitative interviews, extracting the non-pharmacological self-management strategies from the two systematic reviews [34,35] and also from other systematic reviews and RCTs published after the publication of the two systematic reviews, and aligning with the Capability, Opportunity, Motivation and Behaviour (COM-B) model for behaviour change and for long-term adherence to the interventions. To integrate the practice recommendations and the models of care, the Clinical Oncology Society of Australia (COSA) model of survivorship care and Cancer Australia’s optimal care pathways will be used to ensure the interventions align with the current best practices to tailor the unique needs of BCSs with CVDs.

Two qualitative descriptive studies exploring the attitudes, perspectives and experiences of nurses, GPs and allied health professionals who are end users and stakeholders in the management of BCSs (qualitative study one) and the needs, preferences and challenges of BCSs who are end users of this intervention (qualitative study two) will be utilised in the formulation of the preliminary NGPS protocol [44]. As participant recruitment and data collection are ongoing for these two qualitative studies, results are not yet available.

#### 2.1.1. Qualitative Study One: Qualitative Study Insights from Primary Healthcare Professionals

(1) Study sample and setting

Primary healthcare professionals, including practice nurses, GPs and allied health professionals such as physiotherapists, will be recruited from primary care medical centres in Victoria, Australia.

(2) Inclusion and exclusion criteria

Participants will be included if they are: (1) healthcare professionals (e.g., nurses, GPs, allied health professionals); (2) currently practicing in primary care settings; and (3) have a minimum of six months of experience working with or involved in the care and management of BCSs in primary care settings. They will be excluded if they have one of the following: (1) if there is any conflict of interest that might bias the study results; (2) student nurses, student doctors and student allied health professionals; (3) managers who are not working directly with patients or providing direct patient care; (4) healthcare professionals who are planning to end their contract or leave the place of work within the duration of the study.

(3) Sampling, sample size and study procedure

Convenience sampling will be used to recruit participants [45], and sample size will be determined by “data saturation” [46]. Information will be sent via email invitations from the centre reception to recruit eligible healthcare professionals from selected primary care centres in Victoria, Australia. Recruitment will also occur through professional and social media platforms, as well as personal and professional networks. The healthcare professionals at the centre will be introduced to the study through meetings, presentations, or brochures to support recruitment. The potential participants will be contacted through mail, email, over the phone or face-to-face and provided a brief explanation of the study and the inclusion criteria. The interested participants will then be contacted by the researcher (AC) at their convenience and provided with detailed information about the study and asked to obtain written consent. The interviews will be conducted by the primary researcher (AC) using a semi-structured interview guide, which will be piloted with the first two participants to ensure it is clear and comprehensive with possible modifications. The interviews will be audio-recorded, and notes will be taken during the interviews with the participants’ consent.

#### 2.1.2. Qualitative Study Two: Qualitative Study Insights from BCSs with CVDs

BCSs with CVDs will be recruited from primary care medical centres in Victoria.

(1) Inclusion and exclusion criteria

Participants will be included if they: (1) are female patients aged 18 years or older; (2) have a confirmed diagnosis of breast cancer at stage I, II, or IIIa; (3) have a confirmed diagnosis of CVDs, such as heart failure, hypertension, coronary artery disease, cerebrovascular accidents (e.g., stroke, transient ischemic attack), cardiomyopathy and arrhythmias; (4) are able to read, write, and communicate in English; (5) patients who have completed active anticancer treatments, including chemotherapy and radiotherapy; (6) patients who are receiving regular healthcare in primary care from GP or nurses; (7) patients who are willing to participate in the research study and provide informed consent. Patients will be excluded if (1) they have unstable health conditions (e.g., critically ill patients or mental health patients); (2) patients with cognitive impairment (e.g., memory loss, disorientation, personality or mood changes); or (3) currently involved in another research study.

(2) Sampling, sample size and study procedure

A convenience sampling method will be utilised [45], and the sample size will be determined by data saturation [46]. Participants from the primary care centres in Victoria, Australia will be recruited in collaboration with the medical centres and the healthcare professionals after the healthcare professionals are informed about the study through meetings, presentations or brochures. The participants will be informed of the study by the treating doctor or the centre, by using flyers, or through emails sent by the centre reception. Recruitment will also occur through professional and social media platforms. Healthcare professionals will also be invited to refer eligible BCSs and provide them with participant information sheets to distribute. The primary researcher (AC) will then contact the interested participants in person, by phone, or by email and obtain a consent form if they are interested in participating.

The primary researcher will conduct semi-structured interviews using an interview guide via face-to-face, telephone or video meetings at a mutually convenient time. All participants will be completing a demographic questionnaire prior to the interview to collect information such as age, occupation, financial status, stage of BC and type of CVD. The interviews will be audio-recorded, and notes will be taken with the participant’s consent.

#### 2.1.3. Data Analysis

The recorded interviews will be transcribed verbatim prior to analysis by the primary researcher. The transcripts will then be double-checked by one of the other researchers against the audio records to ensure completeness and accuracy. An inductive qualitative thematic analysis will be utilised to analyse the data in six steps as described in [47,48]. The analysis will involve a stepwise approach by identifying codes representing specific information within the data, reviewing and recoding the content where necessary, and arranging multiple codes in a dataset into themes. The themes will then be analysed for internal consistency and validity in relation to the entire data set; themes will be named and quotes will be extracted to represent the themes.

#### 2.1.4. Trustworthiness of the Two Qualitative Studies

To establish rigour and trustworthiness, the researchers will incorporate reflexive practices [49]. Credibility will be achieved using excerpts from the transcripts and the initial analysis findings to increase the trustworthiness of the research [50]. Also, the study setting, the participants’ characteristics, the study process, and data analysis will be detailed in the final report to ensure dependability and auditability [51,52].

#### 2.1.5. Identification of Research Evidence and Relevant Theories

Non-pharmacological self-management strategies from two systematic reviews [34,35] and a synthesis of current literature will be utilised to develop the NGPS intervention. Non-pharmacological self-management interventions for CVDs [35] and non-pharmacological self-management interventions for breast cancer survivors [34] are presented in Table 1 and Table 2 respectively. Further credible literature, such as systematic reviews and RCTs, published after the publication of the two systematic reviews, will be identified in the same databases as the two systematic reviews to warrant the use of the latest evidence for the NGPS intervention development.

The COM-B model, underpinned by behavioural change from the interrelated components of capability (physical and psychological), opportunity (physical and social), and motivation (automatic and reflective), will serve as the primary theoretical framework for the NGPS intervention development [53]. The COM-B model applies an iterative approach. The physical capability will be used to assess the physical and psychological ability of BCS to participate in the self-management interventions and to understand the knowledge and skills associated with self-management. The social opportunity will be used to assess the family and caregiver involvement in the self-management skills, and the physical opportunity will be used to assess the accessibility of self-management interventions. The automatic motivation will assess the usual habits and techniques, and reflective motivation will assess the health goals in the management of CVDs. The NGPS protocol informed by the COM-B model will be able to address the factors influencing self-management behaviours, increasing the likelihood of successful behaviour change and long-term adherence to the intervention protocol.

#### 2.1.6. Identification of Practice Recommendations and Models of Care

Two models of care in Australia, including Cancer Australia’s optimal care pathways for women with breast cancer and the Clinical Oncology Society of Australia (COSA)’s Model of Survivorship Care, will be utilised in the development of the intervention protocol, which aligns with the best practices [54,55]. A layered approach will be employed to incorporate the two models of care into the protocol: (1) adopt the continuity of care principles and the survivorship care domains described by COSA by adopting a multidisciplinary approach involving GPs, nurses, and allied healthcare professionals for patient-centred care, (2) use a patient-centred care plan to address the unique needs and risks of BC and CVDs, (3) incorporate care coordination to ensure the care provided is systematic, connected and timely, and (4) the protocol will promote open communication, trust and shared decision-making between patients and healthcare professionals. This will ensure the interventions align with the current best practice to address the unique needs of the BCSs.

#### 2.1.7. The Preliminary Version of the NGPS Protocol

The findings from the two qualitative studies, together with current research evidence, the above-mentioned theories, current practice recommendations and existing models of care, will be used to develop the preliminary protocol. The preliminary protocol contains four-week nurse-led self-management interventions tailored to the needs of the BCS with CVDs. This preliminary protocol will be reviewed and updated based on the qualitative study findings before the co-design. A copy of the preliminary self-management protocol is presented in Table 3.

#### 2.1.8. Co-Design Workshop(s) and Content Validation of the Preliminary NGPS Protocol

Co-design workshops will be conducted with healthcare professionals and BCSs with CVDs to improve the content, duration, procedure and wording of the revised and updated preliminary protocol. The workshop(s) will discuss the preliminary protocol with BCSs and healthcare professionals. All participants from the qualitative studies will be invited to the co-design workshop. The primary researcher will then revise and update the protocol based on the experts’ and stakeholders’ comments and suggestions. Once all comments and suggestions have been addressed and no further feedback remains, a content validity study will be conducted. Another co-design workshop will be inviting all the participants for the ‘content validity study’ to validate the preliminary protocol by employing a response form with four criteria: representativeness of the content domain, clarity of the item, factor structure and comprehensiveness of the measure, as described by [56]. The experts will rate the scale from one to four for representativeness and clarity (1 = not representative of the domain or clear; 4 = representative or clear), which is demonstrated by the usefulness of the preliminary protocol items to represent the content domain as described in the theoretical definition. The participants will be evaluating the clarity of each item on the same scale from one to four based on how clearly an item is worded. To assess the factorial structure of the protocol, the items will be grouped according to the factor, and the experts will be asked to assess how well the item measures that factor. The last criterion item is to evaluate the comprehensiveness of the measure. The experts will be asked to evaluate the entire measure and indicate the addition or deletion of any item.

Content validity index (CVI), including CVI for item (I-CVI) and CVI for scale (S-CVI), will be measured during the content validation to assess the relevance and clarity of each item in the protocol [57]. Item- and scale-level CVI values of 0.78 and 0.80 respectively, with at least 80% of items rated as relevant, will be considered acceptable [58,59]. Items that fail to reach a satisfactory CVI will be further discussed and refined until consensus has been reached among the panel experts via a group discussion.

### 2.2. Phase II: The Pilot Study and the Qualitative Process Evaluation

Study design of the pilot study

In phase II, the newly developed intervention protocol’s feasibility and acceptability will be tested using a pre-post pilot study incorporating a qualitative process evaluation.

(1) Pilot study: Study sample

The inclusion criteria will be female patients (1) aged 18 years or older; (2) confirmed diagnosis of breast cancer at stage I, II, or IIIa; (3) confirmed diagnosis of CVDs, such as heart failure, hypertension, coronary artery disease, cerebrovascular accidents (e.g., stroke, transient ischemic attack), cardiomyopathy and arrhythmias; (4) able to read, write, and communicate in English; (5) have completed active anticancer treatment including chemotherapy and radiotherapy, and are receiving regular healthcare in primary care centre from GP/practice nurses; (6) willing to participate in the research study and provide informed consent. The exclusion criteria: patients with unstable health conditions with mental health issues or critically ill patients unable to participate in physical activities (e.g., suffering from life-threatening diseases, extremely weak), cognitive impairment, currently involved in another research study, or scheduled anticancer treatment during the study period.

(2) Sample size and study settings

Based on the rule of thumb suggested by Julious [60], a sample size of 12 will be employed in this pilot single-group pre-post study. When taking into account a 20% dropout rate based on a clinical study by Cramer et al. [61], 15 participants will be recruited for this study.

(3) Study procedure

Potential participants will be identified and approached in collaboration with the primary care centres and the healthcare professionals. The participants will be informed of the study by the treating doctor or the centre by using flyers or through emails sent by the centre reception. The healthcare professionals at the centre will be introduced to the study through meetings, presentations or brochures to help with the recruitment and to distribute the participant information sheet. The interested participants will contact the primary researcher. Prior to recruitment, potential participants will be screened for eligibility, and informed consent will be obtained from eligible participants after explaining the study’s purpose, procedures, and risks. Participants will be advised to continue receiving usual care from their medical centre during the study.

The planned data collection will be taking place at three points during the study: at the beginning of the study before the intervention (T1). At week four immediately after the NGPS intervention (T2) and at week 12, which is eight weeks post-intervention (T3). The eight-week follow-up is based on previous research suggesting that it typically takes around eight weeks for individuals to change a habit or behaviour [62]. The interventions will be carried out through a combination of telehealth and in-person consultations, with alternating durations depending on the session type.

A qualitative process evaluation will be conducted at the end of the intervention, at week four by using semi-structured interviews. All the participants will be invited to participate in the qualitative interviews.

#### 2.2.1. Outcomes and Measurements

The study will employ a demographic data sheet that has been specifically developed by the research team to gather information on the socio-demographic characteristics of the patients, such as age, height, race/ethnicity, as well as their medical history, such as date of BC and CVD diagnosis, the current stage of BC, the type and date of BC and CVD treatment.

(1) Primary outcomes

The primary outcomes will be the feasibility, acceptability and safety outcomes. The feasibility outcome will measure the feasibility of the questionnaires, recruitment and the intervention delivery. The acceptability of the intervention will be assessed by assessing the acceptability among the participants. The safety of the intervention will be measured by the safe delivery of the intervention. The primary outcome will also measure the recruitment, retention, and dropout and will be evaluated as the fundamental aim.

1. The recruitment rate is the percentage of eligible participants who consented to participate in the study.
2. The retention rate will measure the percentage of participants who remain in the study throughout the entire study period, from enrolment to follow-up (week 12).
3. The dropout rate will be calculated based on the percentage of participants withdrawn from the study after enrolment.
4. The feasibility of the questionnaires will be assessed by measuring the percentage of missing responses for each item and the entire scale.
5. The feasibility of the recruitment will be assessed by measuring the number of weeks or months needed to achieve the target number of sample size.
6. The adherence rate to the NGPS protocol will measure the percentage of participants who followed the interventions as intended. This will measure the feasibility and acceptability of the study intervention.
7. The safety of the intervention includes discomfort or adverse effects related to NGPS intervention by keeping a daily logbook and reporting via telephone or face-to-face.
8. Acceptability will assess the participant’s satisfaction, benefits received and obstacles faced using an investigator-created questionnaire.

(2) Secondary outcomes

The secondary outcomes will measure the physiological outcomes, which include weight, BMI, blood pressure and heart rate. The secondary outcomes will also evaluate the clinical outcomes, the preliminary effects of the NGPS intervention on various measures, behavioural outcomes, which include dietary outcomes, physical activity and smoking status; psychological outcomes which include self-efficacy, QoL, and anxiety and depression; and lastly healthcare usage.

1. The dietary outcome will be measured using a 15-item Food Frequency Questionnaire (short 15-item FFQ), which has been tested in female patients and widely used in cardiac patients to screen poor dietary patterns and has an overall good agreement with the diet history [63,64].
2. The International Physical Activity Questionnaire-Short Form (IPAQ-SF) is a nine-item self-report tool and is a well-validated tool tested in Australian adults which will be used to assess physical activity, which has good reliability and validity when used in BCS [65,66].
3. The smoking status will be assessed using a self-reported questionnaire.
4. Psychological outcomes include self-efficacy, which will be measured using the General Self-Efficacy Scale (GSES) [67]. The GSES is a 10-item psychometric instrument to measure the participants’ ability to cope with various challenging situations, has good reliability and validity across a range of populations and is recommended for use in pilot studies [67].
5. The QoL will be measured using the Functional Assessment of Cancer Therapy-Breast (FACT-B), a 37-item tool targeted for BC patients, which showed satisfactory reliability and validity [68].
6. The anxiety and depression will be measured using a 14-item Hospital Anxiety and Depression Scale (HADS), which has been widely adopted in cancer patients and in primary care settings and noted to have satisfactory psychometric properties [69].
7. The healthcare usage will be measured using an investigator-created logbook by measuring the number of hospital visits or GP visits for cardiovascular conditions during the phone calls.

#### 2.2.2. Data Analysis of the Pilot Study

Data will be analysed using descriptive and exploratory inferential statistics using SPSS 26.0 with the intention of feasibility assessment and not for formal hypothesis testing [70]. In a single-group pre-post-test study, the characteristics of the sample will be summarised using descriptive statistics such as frequency counts, percentages, means, and standard deviations. The descriptive data analysis can give an overview of the sample’s age, gender, ethnicity, education level, income, breast cancer treatments, and other demographic variables.

The feasibility outcomes of the study such as recruitment, follow-up assessments, and questionnaire/intervention acceptability will be determined using descriptive data [71]. Mean and standard deviation will be used to analyse interval and ratio data, such as the time taken for participant recruitment and the average time and frequency of physical activity [71]. The acceptability of the questionnaires will be determined by calculating the proportion of missing data [71]. The feedback on the intervention is measured by assessing the participants’ satisfaction scores. Any adverse events related to physical activity, such as musculoskeletal aches and pain, dizziness, and falls, will be presented as the absolute number of events and the total percentage.

Secondary clinical and behavioural outcomes will be presented descriptively at each assessment point to evaluate the feasibility outcomes for future randomization [70]. Changes over time will be explored using non-parametric methods, given the small sample size and single-group pre–post design [71,72]. The Friedman test will be conducted for continuous variables to correlate the data at three different time points [73]. Cochran’s Q test will be utilised for categorical variables to assess changes in proportions between the pre-test and post-test assessments [74]. These analyses will be exploratory only and will not be interpreted as formal hypothesis tests. Analyses will be conducted using available data from participants who complete each assessment. The extent and reasons for missing data will be reported. Participant attrition will be monitored throughout the study, and differences between completers and non-completers will be examined to evaluate potential attrition bias. No imputation of missing data is planned due to the small sample size and feasibility focus of this study.

#### 2.2.3. Quality Assurance and Intervention Fidelity

To ensure the quality assurance of the pilot study, the primary researcher will create standard operating procedures for all study procedures, including recruitment and data collection. Participant recruitment will be conducted using inclusion and exclusion criteria, following recruitment procedures detailed in the study protocol, and all recruitment activities will be documented, monitored, and reported at the biweekly research meetings. Data collection will be conducted by the primary researcher who is completely aware of the data collection procedures to ensure standardisation, and the data will be collected at the time points mentioned to ensure quality.

To ensure intervention fidelity, interventions from an intervention strategy pool (the intervention strategy pool will be developed and validated after the qualitative interviews) will be used for standardisation and to avoid variability in the delivery. The researchers will maintain a study activity log, including intervention delivery and any deviations from the protocol. These details will be reported at the biweekly research meetings. Any decisions on altering the study protocol will be discussed between the researchers and a letter of variation will be submitted to the University of Southern Queensland Human Research Ethics Committee, stating the changes and obtaining final approval. Additionally, the primary researcher will obtain feedback from the patients and the healthcare providers through qualitative process evaluation (as described in Section 2.3) on various components of the NGPS intervention such as relevance, accuracy and wording of the intervention protocol to ensure all components are delivered as intended.

### 2.3. Qualitative Process Evaluation of the Pilot Study

To understand the interventions’ strengths and weaknesses, a qualitative process evaluation will be conducted with both patients and healthcare professionals.

This qualitative study will invite all patients and healthcare professionals who participated in the NGPS intervention. The consented participants will be contacted to schedule an interview at a mutually convenient time after the pilot study. Participants will be given the option to choose between a face-to-face interview, a telephone interview or a video meeting, based on their preference. The doctoral researcher will use a semi-structured interview guide, and the interview will be audio recorded and will involve taking field notes. Before obtaining written consent, a brief explanation of the study will be provided and the participants will be reminded that participation is voluntary and they can withdraw from the study at any time. Data analysis and quality assurance will be the same as in the phase I qualitative study.

## 3. Discussion

In this study, the adoption of the MRC framework and co-design framework as guiding frameworks will provide significant methodological strength. The use of the MRC framework provides a structured and evidence-based approach for developing and piloting the NGPS intervention. It will ensure the intervention is grounded in existing evidence and theories to strengthen the methodological rigour and be suitable for real-world implementation. At the same time the integration of the co-design framework provides meaningful engagement of stakeholders such as BCSs with CVDs and primary healthcare professionals. Incorporating stakeholder involvement in complex health intervention design enhances the acceptability and potential effectiveness of the interventions [75,76]. A user-centred design emphasises the needs and interests of end-users, which will increase the use of research results [77]. Together this will strengthen the overall credibility and practical value of the study, ensuring that the intervention is both evidence-based and patient-centred. The research team also considers the potential challenges in the study. The willingness of stakeholders to contribute to the intervention design and testing can be a potential implementation challenge. Additionally, the study’s settings in primary care and limited resources to develop culturally sensitive or multilingual materials may be challenging factors in the inclusivity of the interventions.

Cardiovascular diseases are underexplored despite the growing evidence that BCSs are at high risk for CVDs due to the cardiotoxic effects of the BC treatment as well as the overlapping risk factors between breast cancer and CVDs. Even though cardio-oncology and survivorship programmes exist, they are not addressing the needs and challenges of BCSs with CVDs due to poor coordination between cardiology and oncology and the absence of shared care models [13,28]. This study will contribute by codesigning self-management non-pharmacological interventions for BCSs with CVDs by integrating the latest evidence, theories, and practice standards. The stakeholder involvement in this study will improve the intervention quality by ensuring that content, delivery methods and intervention strategies reflect the experiences and priorities of intended users [78]. Exploring the feasibility and acceptability of the NGPS intervention in primary care settings may inform survivorship care guidelines and will provide primary healthcare professionals such as nurses, GPs, and allied health professionals with evidence-based tools that will promote long-term health outcomes for patients with both BC and CVDs.

This study has several limitations. The study will be conducted in a specific context within the Australian primary care setting, which could limit the generalisability of the results to other settings. Although multiple recruitment channels (e.g., GP referral, primary clinics and social media platforms) will be used to recruit participants in this study, the use of convenience sampling may introduce selection bias, and the findings may not represent the experience and perspectives of all individuals within the target population, limiting the transferability of the results to other settings. No formal sample size calculation was undertaken to test the hypothesis regarding acceptable recruitment and retention rates, as progression criteria were not pre-specified. Instead, the study was exploratory in nature and aimed to estimate the feasibility parameters. The findings will be interpreted as preliminary and descriptive rather than hypothesis-driven. Future studies should incorporate predefined progression criteria and associated hypothesis testing to enable more robust evaluation of recruitment and retention outcomes. This study uses a single-group pre-post-test with a small sample size; hence, the study cannot determine the efficacy of the self-management interventions. Next, the eight-week post-intervention follow-up might be short in duration; however, the primary aim of the study is to assess the feasibility of the intervention and data collection procedures before conducting a future RCT with a longer follow-up. Additionally, it would be challenging to achieve enduring behaviour change among BCSs even though an eight-week follow-up will be conducted as self-management lifestyle interventions require ongoing commitments and lifestyle modifications that may be challenging to maintain. To enhance adherence beyond the scheduled intervention period, future studies can consider including self-monitoring tools, digital reminders, and peer-support mechanisms. Additionally, strategies such as promoting habit formation by linking the desired behaviour to existing daily routines, enhancing intrinsic motivation by placing focus on personal goals, and values rather than external rewards.

## 4. Conclusions

Overall, this proposed study has the potential to lay the groundwork for self-management interventions for BCSs with CVDs, ultimately enhancing long-term survivorship outcomes, lowering healthcare costs, and serving as a tool for primary healthcare.

## Figures and Tables

**Table 1:** Self-management non-pharmacological interventions for CVDs.

Condition	Non-Pharmacological Interventions
CVDs	Diet, physical activity, smoking cessation and weight management
Coronary heart disease	Diet, physical activity, smoking cessation and weight management
Heart failure	Diet and physical activity
Atrial fibrillation	Weight loss and exercise recommended to avoid intensive endurance training
Stroke	Diet, physical activity, smoking cessation, weight management and decreased alcohol consumption and mental health interventions such as mind–body exercise such as taichi, yoga and qigong for mental health management
Peripheral artery disease	Physical activity and smoking cessation
Hypertrophic cardiomyopathy	Moderate to low degree recreational activity and achieve or maintain weight loss for obese individuals

The information presented in this table was adapted from the systematic review [35].

**Table 2:** Self-management non-pharmacological interventions for BCSs.

Condition	Non-Pharmacological Interventions
Anxiety, depression and distress	Meditation, massage therapy, passive music therapy and hypnosis. Self-administered cognitive behavioural stress management for the management of anxiety and progressive muscle relaxation, guided imagery, visualisation techniques, and autogenic training) for depression management
Fatigue	Physical activity, yoga and hypnosis
Pain	Physical therapy, music therapy post-surgery and hypnosis
Breast cancer-related lymphedema	Progressive resistance training, aerobic exercises, aquatic exercises, self-massage, diaphragmatic breathing and weight reduction
Nausea and vomiting	Self-acupressure and relaxation
Neuropathy	Physical activity
Sleep improvement	Yoga
Vasomotor/hot flushes	Physical activity
Quality of life improvement	Regular exercise or sports, meditation, yoga and hypnosis
Reduction in risk of recurrence	Physical activity, exercise and weight management
Health promotion	Weight management, physical activity, nutrition, alcohol limitation, and smoking cessation

The information presented in this table was adapted from the systematic review [34].

**Table 3:** A preliminary NGPS intervention protocol.

W1 Initial nurse-led consultation	Format: in-person Time: 15–30 min Health professionals: nurse, and involve allied health professionals if needed Patients: patient and caregiver	Assess the breast cancer survivors’ knowledge of their cardiac conditions and breast cancer, evaluate health literacy, access to resources and motivation to manage their health condition by using the COM-B model. Using the COM-B model, identify the patients’ needs, preferences, and goals and the factors influencing self-management by evaluating their capability, opportunity and motivation to manage their health effectively. Select suitable intervention strategies from the “intervention strategy pool” and develop personalised care plans based on identified factors and a multidisciplinary approach using the COM-B model.
W1 Self-management session	In-person 45–60 min Nurse, patient, and caregiver Involve GP and allied health professionals if needed	Further detail the overall care plan and teach and/or demonstrate the selected suitable intervention strategies to support the behaviour change in patients and evaluate patients to ensure they have mastered the skills for optimal outcomes. Enhance the patient’s capability by providing an education booklet that promotes explicit understanding and engagement in self-management behaviours.
W2 Telehealth with nurse	Telehealth or in-person 15–30 min Nurse and patient	Nurse-led weekly telehealth support sessions using the COM-B model Monitor progress and the appropriateness of the implemented intervention strategies such as frequency and duration to ensure they effectively support the behaviour change. Provide guidance and support to enhance the patient’s capability. Reassess and adjust the care plan based on ongoing assessments to ensure optimal outcomes. Reinforce behaviour change techniques to enhance motivation. Instruct patients to report any healthcare usage during the intervention to maintain accurate monitoring. Encourage patients to ask questions and seek clarification to improve understanding and engagement.
W3 Telehealth with nurse	Telehealth or in-person 15–30 min nurse, patient, and caregiver	Nurse-consultation Medical evaluation. Address any medical concerns via coordination with the multidisciplinary team (including GP and allied health if needed). Review and update the care plan as needed. Check whether all the intervention strategies were correctly implemented. Nurse-led weekly telehealth support sessions (content the same as week 2).
W4 Telehealth with nurse	Telehealth or in-person 15–30 min Nurse, patient, and caregiver	Nurse-led weekly telehealth support sessions using the COM-B model (contents the same as week 2)

## Data Availability

No new data were created or analyzed in this study. Data sharing is not applicable.

## Public Involvement Statement

No public involvement in any aspect of this research.

## Guidelines and Standards Statement

This manuscript was drafted against the Template for Intervention Description and Replication (TIDieR) checklist alongside Standard Protocol Items: Recommendations for interventional Trials (SPIRIT) for comprehensive description of intervention content feasibility research.

## Abbreviations

The following abbreviations are used in this manuscript

BC	Breast Cancer
BCS/BCSs	Breast Cancer Survivor(s)
CVD/CVDs	Cardiovascular disease(s)
NGPS	Nurse-led GP-supported self-management interventions
BMI	Body Mass Index
AIC	Anthracycline-induced cardiomyopathy
GP	General Practitioner
RCT	Randomised Controlled Study
QoL	Quality of Life
MRC	Medical Research Council
COM-B	Capability, motivation and behaviour
COSA	Clinical Oncology Society of Australia
CVI	Content validity index
I-CVI	Content validity index for item
S-CVI	Content validity index for scale
FFQ	Food Frequency Questionnaire
IPAQ-SF	Physical Activity Questionnaire-Short Form
GSES	General Self-Efficacy Scale
FACT-B	Functional Assessment of Cancer Therapy-Breast
HADS	Hospital Anxiety and Depression Scale
ITT	Intent To Treat
